# Porcine epidemic diarrhea virus promotes viral replication via ROS/HIF-1α-mediated glycolysis

**DOI:** 10.1016/j.redox.2026.104008

**Published:** 2026-01-05

**Authors:** Yafang Xu, Jinqiu Zhang, Chengwei Yin, Laizhen Liu, Zhenglei Wang, Shaodong Fu, Rong Fan, Yanyan Zhao, Jinfeng Miao

**Affiliations:** a College of Veterinary Medicine, Nanjing Agricultural University, Nanjing, 210095, China; b Institute of Veterinary Immunology & Engineering, Jiangsu Academy of Agricultural Sciences, Nanjing, 210014, China; c Engineering Research Center for the Prevention and Control of Animal Original Zoonosis & College of Life Science, Longyan University, Longyan, 364012, China; d College of Veterinary Medicine, Xinjiang Agricultural University, Urumqi, 830052, China; e College of Veterinary Medicine, Shanxi Agricultural University, Jinzhong, 030801, China

**Keywords:** Porcine epidemic diarrhea virus, HIF-1α, Glycolysis, Mitochondria, ROS, Metabolism

## Abstract

Porcine epidemic diarrhea virus (PEDV), a highly pathogenic coronavirus, causes recurrent outbreaks of severe enteric disease, posing a significant threat to the global swine industry. The persistent challenge highlights the urgent need for a deeper understanding of host-virus interactions to improve prevention and control strategies. Here, we demonstrated that PEDV infection reprogrammed host metabolism toward aerobic glycolysis, a metabolic shift that not only facilitated viral replication but also established an immunosuppressive microenvironment. PEDV infection activated the hypoxia-inducible factor-1α (HIF-1α) pathway and induced mitochondrial dysfunction, leading to the accumulation of mitochondrial reactive oxygen species (mROS), which in turn stabilized HIF-1α, creating a positive feedback loop that amplified glycolytic gene expression and lactate production. We confirmed that glycolysis was essential for PEDV replication, and that elevated glucose levels enhanced replication efficiency. Furthermore, PEDV-induced glycolysis and lactate accumulation inhibited the generation of interferons (IFNs), thereby facilitating immune evasion. Collectively, our findings revealed a metabolic-immune axis exploited by PEDV to optimize viral replication and subvert host defenses. This study not only provides novel insights into the metabolic adaptations underlying PEDV pathogenesis but also highlights host metabolic pathways as potential therapeutic targets to combat PEDV and other related coronaviruses.

## 1 Introduction

PEDV remains a major etiological agent responsible for severe enteric disease with high mortality in neonatal piglets, causing substantial economic losses to the global swine industry [1,2]. Despite the widespread use of vaccines and enhanced biosecurity measures, porcine epidemic diarrhea (PED) outbreaks continue to recur, underscoring the limited effectiveness of current control strategies [3,4]. This persistent challenge highlights the urgent need for a deeper understanding of host-virus interactions that govern viral replication and pathogenesis, which may ultimately facilitate the development of novel antiviral strategies.

Viruses are obligate intracellular parasites that need to utilize the energy of host cells or hijack cellular metabolic resources to support the rapid proliferation and assembly of progeny virus particles [5]. Accordingly, viral infection frequently interferes with host metabolic pathways and triggers remodeling of host central carbon metabolism (CCM)—including glycolysis, the pentose phosphate pathway (PPP), and the tricarboxylic acid (TCA) cycle—to reallocate bioenergetic and biosynthetic resources [6,7]. A common metabolic reprogramming during viral infection is aerobic glycolysis (the Warburg effect), typically characterized by enhanced glucose uptake and lactate production, and reported for diverse viruses such as severe acute respiratory syndrome coronavirus 2 (SARS-CoV-2), dengue virus, hepatitis C virus (HCV), and human papillomavirus (HPV) [[8], [9], [10], [11], [12]]. Nevertheless, some viruses also display heterogeneous metabolic strategies, with glycolytic and mitochondrial pathways being coordinately rewired in a time- and cell type-dependent manner [[13], [14], [15], [16], [17]]. Importantly, metabolic remodeling is not merely a bystander consequence of infection; it can directly shape antiviral signaling and inflammatory responses, thereby influencing viral fitness and disease pathophysiology [18,19]. However, to our knowledge, how PEDV rewires host cellular metabolism, and how such remodeling feeds back onto antiviral defenses, remains incompletely understood.

Mitochondria are a key hub linking metabolism, redox homeostasis, and innate immune signaling. Mitochondrial dysfunction frequently results in excessive production of reactive oxygen species (ROS), which can function as signaling molecules to regulate transcriptional programs and immune responses [20,21]. HIF-1α, a redox-responsive transcription factor and master regulator of glycolytic gene expression, can be stabilized by mROS and has been implicated in viral replication and immune modulation in several viral systems [[22], [23], [24], [25]]. For example, SARS-CoV-2 ORF3a protein promoted HIF-1α production through inducing mitochondrial damage and mROS production, and HIF-1α signaling, immune response, and metabolism pathways are dysregulated in SARS-CoV-2-infected cells [23,24]. In addition, beyond meeting the demands of bioenergy and biosynthesis, accumulating evidence indicates that glycolytic remodeling and its by-products lactate can directly modulate RIG-I–MAVS signaling and IFNs production [[26], [27], [28]]. PEDV has been reported to induce ROS generation and suppress IFNs production [29,30], however, whether PEDV engages a ROS/HIF-1α pathway to rewire host metabolism and create an immunosuppressive intracellular environment remains unclear.

In this study, we demonstrated that PEDV infection induced mitochondrial dysfunction and mROS accumulation, resulting in the stabilization of HIF-1α and a shift toward enhanced glycolysis. We further found that increased glycolytic activity and lactate accumulation promoted PEDV replication while suppressing type I and type III IFNs responses. Intriguingly, pharmacological or genetic disruption of this redox-metabolic circuit attenuated viral replication. Taken together, our findings elucidate the role of ROS/HIF-1α-mediated glycolysis during PEDV infection and highlight potential metabolism-targeted host pathways for therapeutic intervention.

## 2 Materials and methods

### 2.1 Reagents and antibodies

2-(7-Nitro-2,1,3-benzoxadiazol-4-yl)-d-glucosamine (2-NBDG; S8914), 2-Deoxy-d-glucose (2-DG; S4701), and Sodium oxamate (Oxa; S6871) were purchased from Selleck Chemicals (China). KC7F2 (HIF-1α inhibitor, HY-18777), dimethyloxalylglycine (DMOG, HIF-1α activator, HY-15893), *N*-Acetyl-l-cysteine (NAC; HY-B0215), Rotenone (ROT; HY-B1756), and Carbonyl cyanide 3-chlorophenylhydrazone (CCCP; HY-100941) were purchased from MedChemExpress (MCE, China). (L)-Sodium lactate (Lac; T4103) was purchased from TargetMol (China). Phospho-DNMEL (Ser616) Antibody (P-DNMEL (S616); AF8470) was purchased from Affinity Biosciences LTD (China). DyLight 488 Conjugated AffiniPure Goat Anti-mouse IgG (H + L) was purchased from BOSTER Biological Technology (China). Tubulin β polyclonal antibody (β-tubulin; AP0064) and mitochondrial dynamin 1-like protein (Drp1/DNM1L) polyclonal antibody (DNM1L; BS7390) were purchased from Bioworld (China). Glucose Transporter 1 (GLUT1) polyclonal antibody (GLUT1; 21829-1-AP), Hexokinase 2 polyclonal antibody (HK2; 22029-1-AP), phosphofructokinase (PFKM) polyclonal antibody (PFKM; 55028-1-AP), 6-phosphofructo-2-kinase (PFKFB3) polyclonal antibody (PFKFB3; 13763-1-AP), and lactate dehydrogenase A (LDHA)-specific polyclonal antibody (LDHA; 19987-1-AP) were purchased from Proteintech (China). Mitofusin-2 (D2D10) Rabbit mAb (Mfn2; 9482), 5′-AMP-activated protein kinase catalytic subunit alpha (AMPKα) antibody (AMPKα; 2532), Phospho-AMPKα (Thr172) (40H9) Rabbit mAb (*p*-AMPK; 2535), mTOR Antibody (mTOR; 2972 S), and Phospho-mTOR (Ser2448) (D9C2) XP® Rabbit mAb (*p*-mTOR; 5536) were purchased from Cell Signaling Technology (USA). MitoSOX™ Red Mitochondrial Superoxide Indicator was purchased from ThermoFisher Scientific (USA). Lactic Acid (L-LA) Content Assay Kit (D799099) was purchased from Sangon Biotech (China). Enhanced mitochondrial membrane potential assay kit with JC-1 (JC-1; C2003S), Enhanced Cell Counting Kit-8 (CCK-8) assay kit (C0041) and ATP assay kit (S0026) were purchased from Beyotime (China). Fetal bovine serum (FBS), Dulbecco's modified Eagle's medium (DMEM), DMEM without d-Glucose and Sodium Pyruvate, and 0.25 % trypsin were purchased from Gibco (USA). Anti-PEDV-N mAb was kindly provided by Dr. Li Bin from the Institute of Veterinary Medicine, Jiangsu Academy of Agricultural Sciences [31].

### 2.2 Cell culture, virus and treatment

Swine testis (ST) cells and Vero cells were purchased from the American Type Culture Collection (ATCC) and stored in our laboratory. Intestinal porcine epithelial cell line IPEC-J2 was kindly provided by Dr. Li Bin from the Institute of Veterinary Medicine, Jiangsu Academy of Agricultural Sciences. Cells were cultured in DMEM supplemented with 10 % FBS at 37 °C in a humidified incubator with 5 % CO_2_. PEDV JS2013, an epidemic strain isolated in China, was stored in our laboratory. The virus preparations were titrated and stored at −80 °C.

Cells were infected with PEDV at a multiplicity of infection (MOI = 1) for 2 h. Then, the virus inoculum was removed, and cells were washed and replenished with serum-free DMEM supplemented with 5 μg/mL trypsin for 24 h or indicated durations. As a mock infection, cells were treated with serum-free DMEM supplemented with 5 μg/mL trypsin.

Prior to different pharmacological treatments, cell viability was determined using the CCK-8 assay kit (Beyotime, Shanghai, China) according to the manufacturer's protocol. Briefly, cells were seeded in 96-well plates at a density of 1 × 10^4^ per well and allowed to adhere overnight. Then, cells were treated with different concentrations of KC7F2, DMOG, ROT, NAC, 2-DG, Oxa, or lactate for indicated durations. Cells treated with PBS or DMSO served as controls. Following treatment, 10 μL of CCK-8 solution was added to each well and cells were incubated for 2 h at 37 °C. The absorbance at 450 nm was measured using a microplate reader (BioTek, USA). Cell viability was calculated as the percentage of absorbance in treated wells relative to control wells.

For inhibitor or activator experiments, cells were treated under the following conditions prior to PEDV challenge: pre-incubation with 40 μM KC7F2 (HIF-1α inhibitor; Selleck Chemicals) for 24 h; exposure to 50 μM DMOG (HIF-1α activator; Selleck Chemicals) for 24 h; treatment with 10 μM ROT (ROS inducer; MCE) for 1 h; or pre-incubation with 6 mM NAC (ROS scavenger; MCE) for 4 h, respectively. For the glycolytic metabolites experiments, cells were treated with the following compounds, 10 mM lactate (TargetMol), 10 mM 2-DG (glycolysis inhibitor; Selleck Chemicals), or 10 mM Oxa (lactate dehydrogenase (LDH) inhibitor; Selleck Chemicals), respectively, at 37 °C for 4 h. After treatment, cells were infected with or without PEDV for another 24 h. Then, the supernatant and cells were collected separately and stored at −80 °C until further analysis.

### 2.3 Metabolomics analysis

PEDV-infected or mock-infected ST cells were collected at 24 h post infection (hpi), respectively, by centrifugation at 1200 rpm for 5 min. For metabolites extraction, 450 μL extract solution (acetonitrile: methanol: water = 2: 2: 1) containing isotopically-labeled internal standard mixture was added to the cell precipitates. After 30 s vortex, the samples were freezed and thawed with liquid nitrogen for 3 times. Then the samples were sonicated for 10 min in ice-water bath. After that, the samples were incubated at −40 °C for 1 h and centrifuged at 12,000 rpm for 15 min at 4 °C. The resulting supernatant was transferred to a fresh glass vial for liquid chromatograph-mass spectrometer (LC/MS) analysis. For targeted metabolomic analysis, four independent biological replicates were included for each group. The quality control (QC) sample was prepared by mixing an equal aliquot of the supernatants from all of the samples.

LC-MS analysis was performed using an UHPLC system (Vanquish, Thermo Fisher Scientific) with a UPLC BEH Amide column (2.1 mm × 100 mm, 1.7 μM) coupled to Q Exactive HFX mass spectrometer (Orbitrap MS, Thermo). The mobile phase consisted of 25 mM ammonium acetate and 25 mM ammonia hydroxide in water (pH = 9.75) and acetonitrile. The auto-sampler temperature was 4 °C, and the injection volume was 3 μL. The QE HFX mass spectrometer was used for its ability to acquire MS/MS spectra on information-dependent acquisition (IDA) mode in the control of the acquisition software (Xcalibur, Thermo). In this mode, the acquisition software continuously evaluates the full scan MS spectrum. The ESI source conditions were set as following: sheath gas flow rate as 30 Arb, Aux gas flow rate as 25 Arb, capillary temperature 350 °C, full MS resolution as 60,000, MS/MS resolution as 7500, collision energy as 10/30/60 in NCE mode, spray Voltage as 3.6 kV (positive) or −3.2 kV (negative), respectively. The different metabolites were identified according to the KEGG metabolome database and the metabolic pathways involved were analyzed using Metabo Analyst.

### 2.4 Quantitative real time PCR (qRT-PCR)

Cells were washed twice with PBS and lysed using RNAiso reagent according to the manufacturer's instructions (Takara, Dalian, China). Total RNA was then reverse transcribed into cDNA using the PrimeScriptTM RT reagent kit (Takara, Dalian, China). The PCR reaction was performed in a total volume of 20 μL using SYBR Premix Ex TaqTM (Takara, Dalian, China), with 2 μL of cDNA as the template. The primer sequences used were outlined in Table S1. As a control, β-actin was amplified simultaneously using a specific pair of primers. Relative gene expression levels were analyzed using the 2^−ΔΔCT^ method.

### 2.5 Western blot assay

The cells were lysed on ice for 30 min in a cell lysis mixture containing cell lysis solution, 20 × protease inhibitor, 20 × protease-phosphatase inhibitor, and 100 × PMSF. The total proteins were denatured and separated by 10 %–15 % sodium dodecyl sulfate polyacrylamide gel electrophoresis (SDS-PAGE). Subsequently, the proteins were transferred onto polyvinylidene fluoride (PVDF) membranes (Millipore, USA) at 90 V for 100 min. The membranes were then blocked with 5 % bovine serum albumin (BSA) and probed with primary antibodies overnight at 4 °C, followed by incubation with the corresponding secondary antibodies for 2 h at room temperature. Protein bands were visualized using enhanced chemiluminescence (Tanon, Shanghai, China) and quantified using ImageJ software (National Institutes of Health, Bethesda, MD, USA).

### 2.6 Glucose uptake assay

The glucose uptake in cultured cells was measured using a 2-NBDG glucose uptake assay kit according to the manufacturer's instructions. Briefly, ST cells were infected with PEDV and incubated for 0, 12, or 24 h under standard culture conditions (37 °C, 5 % CO_2_). At the indicated time points, DMEM was replaced with a glucose-free medium, and cells were incubated for another 30 min. Next, 100 μM of 2-NBDG was added and reacted for 30 min. Finally, the cells were collected and analyzed via a flow cytometer (Beckman Coulter CytoFLEX, Brea, CA, USA).

### 2.7 Measurements of lactic acid production

ST cells were infected with PEDV for 24 h. Cells were harvested by centrifugation at 1000 prm for 5 min at 4 °C, and then sonicated for 10 min in ice-water bath. The lysate was centrifuged at 12,000 rpm for 10 min at 4 °C, and the lactic acid concentration in the supernatant was determined using a lactic acid assay kit (Sangon Biotech, China) according to the manufacturer's instructions. The absorbance values were measured using a microplate reader (Tecan Group Ltd., Männedorf, Switzerland) at 570 nm.

### 2.8 Detection of mitochondrial membrane potential (MMP)

MMP was detected using JC-1 (Beyotime; C2003S) according to the manufacturer's instructions. Briefly, ST cells were infected with PEDV for 24 h. Then, cells were rinsed twice with cold PBS (pH 7.4) and incubated with JC-1 staining solution for 30 min at 37 °C in the dark. After incubation, cells were washed twice with cold PBS to remove excess dye, and the fluorescence intensity was measured using a fluorescence microscope (Thermo Fisher Scientific, Waltham, MA, USA) at the excitation/emission wavelengths of 490/530 nm for the green signal (JC-1 monomer) and 525/590 nm for the red signal (JC-1 aggregates). The fluorescence ratio of red to green was used to assess the MMP level.

### 2.9 Detection of adenosine triphosphate

The adenosine triphosphate (ATP) content in cells was quantified using an ATP assay kit (Beyotime; S0026) according to the manufacturer's instructions. Briefly, ST cells were infected with PEDV for 24 h. The cells were then washed twice with cold PBS (pH 7.4) and processed using the cell lysis buffer provided in the ATP assay kit, followed by centrifugation at 12,000 rpm for 10 min at 4 °C. The supernatant was collected and the ATP concentration was measured using the ATP detection reagent. For the assay, 20 μL of the supernatant was mixed with 100 μL of the ATP assay working solution in a 96-well plate. The luminescence was measured immediately using a microplate reader (Tecan Group Ltd., Männedorf, Switzerland) at an emission wavelength of 560 nm.

### 2.10 Transmission electron microscopy (TEM)

PEDV-infected or mock-infected cells at 24 hpi were collected and washed twice with cold PBS (pH 7.4). The cells were then fixed with 2.5 % glutaraldehyde (Sigma; G5882) at 4 °C for 4 h. Following fixation, the cells were rinsed twice with PBS and serially dehydrated using acetone and embedded in epoxy resin (Sigma; SLCJ5080). Ultrathin sections were prepared and collected onto copper grids. The grids were then stained with uranyl acetate and lead citrate for 10 min at room temperature. Finally, the samples were examined via TEM (JEOL Ltd., Tokyo, Japan) at an accelerating voltage of 80 kV.

### 2.11 Determination of mROS

mROS levels were measured using MitoSOX Red Mitochondrial Superoxide Indicator (Thermo Fisher Scientific). Briefly, ST cells were treated with or without NAC for 4 h and then infected with PEDV for 24 h. Then, cells were incubated in DMEM containing 5 μM MitoSOX Red at 37 °C for 30 min in the dark. After incubation, cells were washed twice with warm PBS to remove excess dyes. For fluorescence microscopy, stained cells were immediately imaged using a fluorescence microscope (Thermo Fisher Scientific, Waltham, MA, USA). For flow cytometric analysis, cells were collected and analyzed using a CytoFLEX flow cytometer (Beckman Coulter, Brea, CA, USA). mROS levels were quantified as mean fluorescence intensity.

### 2.12 Immunofluorescence assay (IFA)

ST cells were infected with PEDV for 24 h. Then, cells were washed with PBS and fixed with 4 % paraformaldehyde for 30 min at room temperature. After fixation, the cells were permeabilized using 0.1 % Triton X-100 (Beyotime, China) for 20 min at room temperature, followed by incubation with 5 % bovine serum albumin for 1 h at 37 °C. Subsequently, the cells were stained with the indicated antibodies overnight at 4 °C and then treated with the corresponding fluorescent secondary antibodies for 1 h at 37 °C. Nuclear DNA was labeled with 4′,6-diamidino-2-phenylindole (DAPI) (Solarbio, Beijing, China) for 10 min. After each step, the cells were washed three times. Finally, slides were subsequently mounted using glycerin and were visualized under a confocal microscope (Olympus, Tokyo, Japan).

### 2.13 Small interfering RNA or overexpression plasmid transfection

Small interfering RNA (siRNA) oligonucleotides targeting endogenous HK2 and LDHA, as well as the LDHA overexpression plasmid (LDHA-OE), were constructed by GenePharma (Shanghai, China). Cells were transfected with siRNA targeting HK2 or LDHA, or with the LDHA-OE plasmid, using Lipofectamine 3000 reagent (Invitrogen) for 6 h. After transfection, the cells were washed three times with PBS and further incubated for 18 h before virus infection.

### 2.14 Statistical analysis

Statistical analyses were performed using GraphPad Prism 9 software. All data were presented as mean ± standard error of the mean (SEM) from three independent biological replicates, unless otherwise stated. For comparisons between two groups, a two-tailed unpaired Student's t-test was applied. For experiments involving more than two groups, one-way analysis of variance (ANOVA) followed by a Tukey's multiple-comparisons test was used, and *P* values of less than 0.05 were considered statistically significant. ∗*P* < 0.05, ∗∗*P* < 0.01.

## 3 Results

### 3.1 PEDV infection reprograms host metabolism toward aerobic glycolysis

**Fig. 1:**
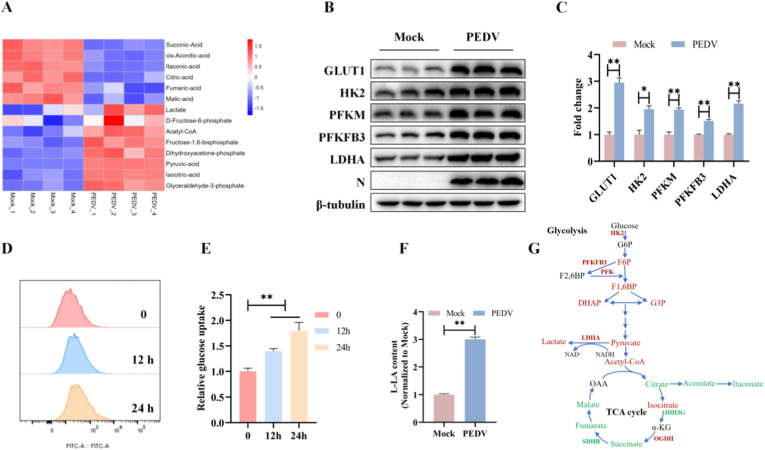
PEDV infection reprograms host metabolism toward aerobic glycolysis. ST cells were infected with PEDV at an MOI of 1 for 24 h. (A) Metabolic heatmap showing relative abundance of glycolytic and TCA cycle metabolites in mock- and PEDV-infected cells. Metabolomic data were obtained from four biological replicates per group. Metabolite levels were normalized to mock controls. (B–C) Protein expression of key glycolytic regulators (GLUT1, HK2, PFKM, PFKFB3, and LDHA) and PEDV N protein was analyzed by western blots. β-Tubulin was used as a control. (D–E) Glucose uptake was measured by flow cytometry and quantified as mean fluorescence intensity (MFI). (F) Lactate production was quantified using a lactate assay kit at 24 hpi. (G) Schematic illustration of glucose metabolic reprogramming during PEDV infection, with upregulated metabolites indicated in red and downregulated metabolites in green. Data were presented as mean ± SEM from three independent experiments. ∗*P* < 0.05, ∗∗*P* < 0.01.

Viruses rely on host metabolic resources to support viral replication, often resulting in extensive metabolic reprogramming of infected cells. To determine whether and how PEDV infection alters host cellular metabolism, we performed targeted metabolomic analysis focusing on glycolysis and the TCA cycle. The data showed that PEDV infection induced a pronounced metabolic shift characterized by enhanced glycolysis and suppression of mitochondrial TCA cycle activity. Multiple glycolytic intermediates, including fructose-6-phosphate, fructose-1,6-bisphosphate, dihydroxyacetone phosphate, pyruvate, lactate, and acetyl-CoA, were significantly increased following infection, whereas key TCA cycle metabolites, such as citrate, aconitate, fumarate, malate, and itaconate, were markedly reduced compared with mock-infected cells (Fig. 1A and Fig. S1A and B). These metabolic changes suggested a shift of glucose metabolism away from mitochondrial oxidation toward glycolysis.

Consistent with the metabolomic findings, PEDV infection significantly upregulated the expression of key glycolytic regulators, including GLUT1, HK2, PFKM, PFKFB3, and LDHA, at both the mRNA and protein levels (Fig. 1B and C and Fig. S1C). In addition, glucose uptake was significantly enhanced in PEDV-infected ST cells, as determined by flow cytometric analysis (Fig. 1D and E), and lactate production was significantly elevated at 24 hpi, further supporting the induction of aerobic glycolysis (Fig. 1F).

Collectively, these results demonstrated that PEDV infection reshaped host cellular metabolism, characterized by a reprogramming of glucose metabolism towards aerobic glycolysis.

### 3.2 PEDV infection activates HIF-1α signaling to promote viral replication

**Fig. 2:**
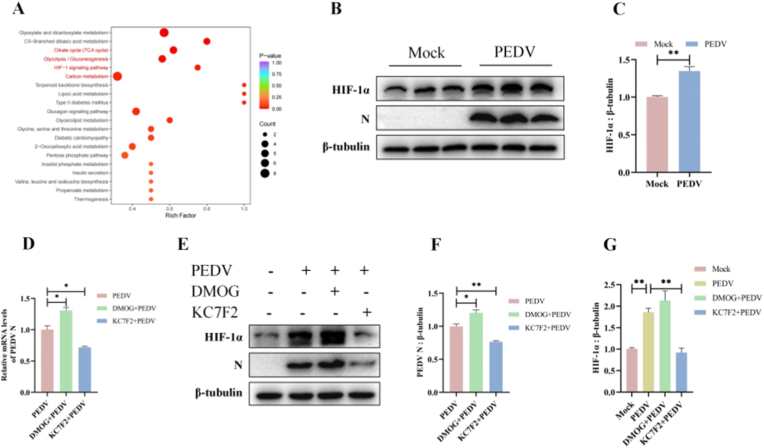
PEDV infection activates HIF-1α signaling to promote viral replication. (A) KEGG pathway enrichment analysis of differential metabolites was identified by targeted metabolomics. The top 20 enriched pathways were shown based on P-values. (B–C) The expression of HIF-1α and PEDV N protein in mock- and PEDV-infected ST cells were determined by western blots. β-Tubulin was used as a control. (D) ST cells were treated with the HIF-1α activator DMOG (50 μM) or the HIF-1α inhibitor KC7F2 (40 μM), respectively, for 24 h, followed by PEDV infection for another 24 h. Viral RNA levels of PEDV N were quantified by qRT-PCR. (E–G) Protein levels of HIF-1α and PEDV N were determined by western blots in cells treated as described in (D). Data were presented as mean ± SEM from three independent experiments. ∗*P* < 0.05, ∗∗*P* < 0.01.

To further explore the signaling pathways associated with PEDV-induced metabolic reprogramming, differential metabolites identified by targeted metabolomics were subjected to KEGG pathway enrichment analysis using the MetaboAnalyst platform. Pathways with significant enrichment were mapped to the swine-specific KEGG database. Among the top enriched pathways, glycolysis, carbon metabolism, the TCA cycle, and the HIF-1α signaling pathway were prominently represented (Fig. 2A), suggesting that HIF-1α signaling may contribute to PEDV-induced metabolic remodeling.

HIF-1α is a central transcriptional regulator of glycolysis, controlling the expression of glucose transporters and key glycolytic enzymes. To determine whether HIF-1α was involved in PEDV-induced metabolic reprogramming, we examined HIF-1α expression following viral infection. Both qRT-PCR and immunoblot analyses revealed a significant increase in HIF-1α mRNA and protein levels in PEDV-infected cells compared with mock-infected controls (Fig. 2B and C and Fig. S2). The elevation of HIF-1α expression was consistent with the upregulation of HIF-1α target genes, including GLUT1 and LDHA, observed in PEDV-infected cells (Fig. 1B and C and Fig. S1C), supporting the involvement of HIF-1α in glycolytic activation during infection.

To further elucidate the role of HIF-1α in PEDV replication, ST cells were treated with the HIF-1α activator DMOG or the HIF-1α inhibitor KC7F2, respectively, prior to viral infection. Pharmacological activation of HIF-1α markedly increased PEDV N protein expression, whereas inhibition of HIF-1α significantly suppressed viral protein accumulation (Fig. 2D–G). Together, these results demonstrated that PEDV infection induced HIF-1α accumulation, which in turn facilitated viral replication.

### 3.3 PEDV infection impairs cellular energy production and induces mitochondria dysfunction

**Fig. 3:**
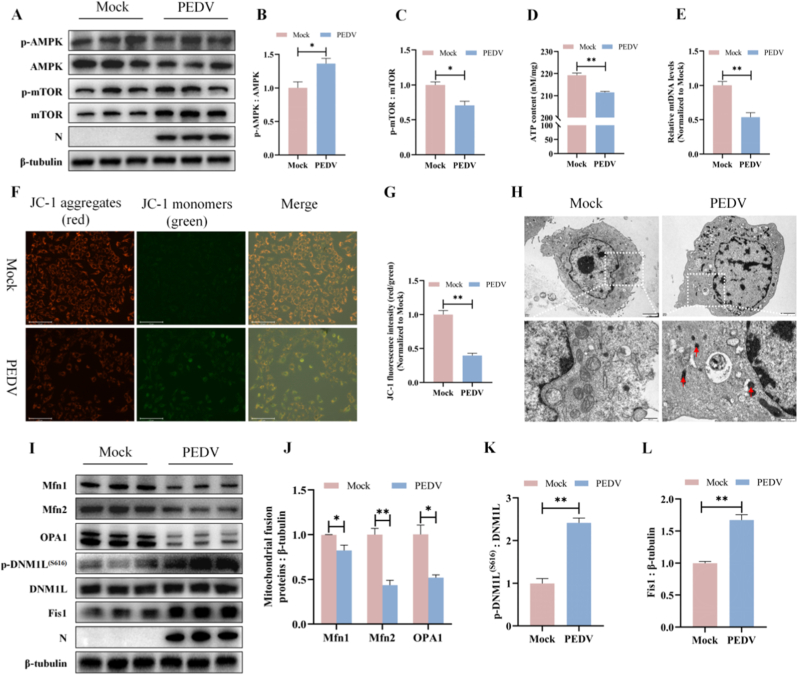
PEDV infection impairs cellular energy production and induces mitochondria dysfunction. (A–C) Protein levels of AMPK, *p*-AMPK, mTOR, and *p*-mTOR in mock- and PEDV-infected ST cells were determined by western blots. β-Tubulin was used as a control. (D) Intracellular ATP levels were measured in ST cells with or without PEDV infection. (E) MtDNA copy number was quantified by q-PCR. (F) MMP was detected by JC-1 staining using fluorescence microscopy. Scale bar: 125 μm. (G) Quantification of JC-1 fluorescence intensity ratios (red/green) corresponding to (F). (H) Representative TEM images showing mitochondrial ultrastructure in mock- and PEDV-infected ST cells. (I–L) Expression levels of Mfn1, Mfn2, OPA1, DNM1L, *p*-DNM1L (S616), and Fis1 were detected by western blots. PEDV N protein was used as an indicator of virus infection. Data were presented as mean ± SEM from three independent experiments. ∗*P* < 0.05, ∗∗*P* < 0.01.

Central carbon metabolism (CCM) is the primary source of intracellular energy, and its disruption is frequently accompanied by altered cellular energy homeostasis. To assess the impact of PEDV infection on cellular energy status, we examined ATP production and the activity of key energy-sensing pathways. PEDV infection markedly increased AMPK phosphorylation while concomitantly reducing mTOR phosphorylation, indicating activation of energy stress signaling (Fig. 3A–C). Consistently, intracellular ATP levels were significantly reduced in PEDV-infected cells (Fig. 3D), suggesting impaired cellular energy production.

Given the central role of mitochondria in ATP generation, we next evaluated mitochondrial integrity and function following PEDV infection. Quantitative PCR analysis revealed a significant reduction in mitochondrial DNA (mtDNA) copy number, indicative of compromised mitochondrial content (Fig. 3E). In parallel, assessment of mitochondrial membrane potential (MMP) using the JC-1 probe showed a pronounced increase in green fluorescence and a decrease in red fluorescence, reflecting mitochondrial depolarization in PEDV-infected cells (Fig. 3F and G).

Ultrastructural examination by TEM further demonstrated that PEDV infection induced profound mitochondrial morphological abnormalities, including reduced mitochondrial volume, disrupted cristae organization, and increased matrix electron density (Fig. 3H). Mitochondrial morphology is tightly regulated by the balance between fusion and fission. Accordingly, we examined the expression of key regulators of mitochondrial dynamics. PEDV infection significantly decreased the levels of the fusion-related proteins mitofusin 1 (Mfn1), mitofusin 2 (Mfn2), and optic atrophy 1 (OPA1), while increasing the expression of the fission-associated protein fission 1 (Fis1) and the phosphorylation of dynamin-related protein 1 (DNM1L) at Ser616 (Fig. 3I–L).

Taken together, these findings indicated that PEDV infection induced a metabolic energy deficit accompanied by mitochondrial depolarization, structural damage, and a shift in mitochondrial dynamics toward fission, ultimately resulting in mitochondrial dysfunction and reduced cellular energy production.

### 3.4 mROS-dependent stabilization of HIF-1α favors PEDV replication

**Fig. 4:**
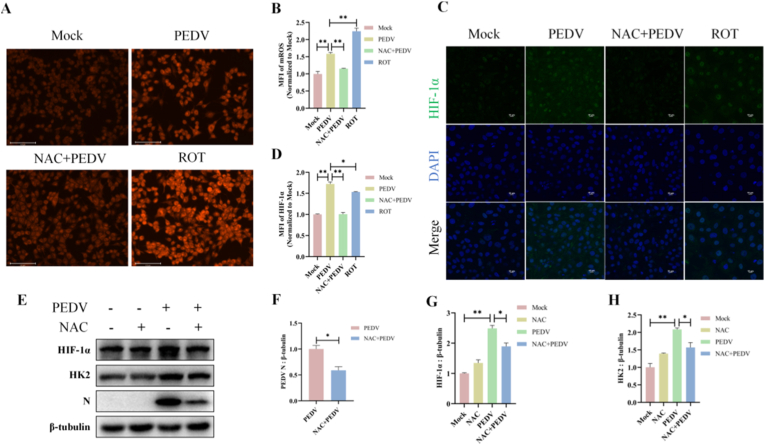
mROS-dependent stabilization of HIF-1α favors PEDV replication. ST cells were treated with or without NAC for 4 h prior to PEDV infection for 24 h. (A) mROS was detected by fluorescence microscopy following MitoSOX staining. Scale bar: 125 μm. (B) Quantification of mROS fluorescence intensity corresponding to (A). (C) HIF-1α in PEDV-infected cells after different treatments was detected by laser confocal microscopy. Scale bar: 10 μm. (D) Quantification of HIF-1α fluorescence intensity corresponding to (C). (E–H) Protein levels of HIF-1α, HK2, and PEDV N were detected by western blots. Data were presented as mean ± SEM from three independent experiments. ∗*P* < 0.05, ∗∗*P* < 0.01.

Mitochondrial dysfunction is commonly accompanied by elevated ROS production, which has been identified as potent inducers of HIF-1α. Considering that PEDV infection activated HIF-1α and induced mitochondrial damage, here we further examined intracellular mROS accumulation upon infection. We observed significant augmentation of mROS in PEDV-infected ST cells, as measured by flow cytometry and fluorescence microscope (Fig. 4A and B and Fig. S3A and B). In contrast, treatment with NAC, a specific antioxidant, effectively suppressed PEDV-induced mROS accumulation, indicating that mROS production was a prominent feature of PEDV infection.

We next investigated whether mROS contributed to HIF-1α stabilization during PEDV infection. It showed that PEDV infection resulted in a pronounced elevation and nuclear localization of HIF-1α, whereas NAC treatment significantly depleted HIF-1α accumulation (Fig. 4C and D). These results indicated that PEDV infection increased cellular HIF-1α levels via a mechanism dependent on elevated mROS levels. In addition, NAC treatment significantly reduced the expression of HK2, the downstream glycolytic target of HIF-1α, accompanied by a substantial decrease in PEDV N protein levels (Fig. 4E–H and Fig. S3C–E). Collectively, these findings suggested that PEDV-induced mROS accumulation promoted the stabilization and elevation of HIF-1α, which in turn enhanced glycolytic activation and viral replication.

### 3.5 Glycolysis is essential for PEDV replication and elevated glucose levels enhance PEDV replication

**Fig. 5:**
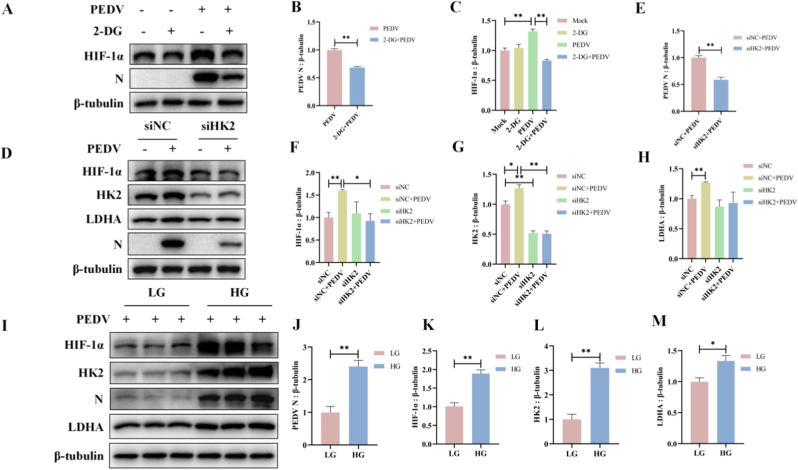
Glycolysis is essential for PEDV replication and elevated glucose levels enhance PEDV replication. (A–C) ST cells were treated with 2-DG for 4 h prior to PEDV infection for 24 h. Protein levels of N and HIF-1α were detected by western blots. (D–H) ST cells were transfected with negative control siRNA (siNC) or HK2-targeting siRNA (siHK2) for 24 h, and then infected with PEDV for 24 h. Protein levels of PEDV N, HIF-1α, HK2, and LDHA were detected by western blots. (I–M) ST cells were exposed to high (25 mM) or low (5 mM) glucose concentrations for 4 h, and then infected with PEDV for 24 h. Protein levels of PEDV N, HK2, HIF-1α, and LDHA were detected by western blots. Data were presented as mean ± SEM from three independent experiments. ∗*P* < 0.05, ∗∗*P* < 0.01.

Given the metabolic reprogramming induced by PEDV infection and the involvement of HIF-1α in regulating glycolytic processes, we investigated whether glycolysis was required for PEDV replication. Pharmacological inhibition of glucose flux with 2-DG markedly reduced PEDV replication, as reflected by decreased PEDV N mRNA and protein levels. In parallel, 2-DG treatment significantly reduced HIF-1α expression at both the transcriptional and protein levels (Fig. 5A–C and Fig. S4A and B).

To further dissect the role of glycolysis in PEDV replication, ST cells were treated with siHK2 to inhibit the glycolytic pathway (Fig. S4C). Knockdown of HK2 significantly reduced the mRNA level of PEDV N gene (Fig. S4D), and markedly decreased the protein expression of PEDV N, HK2, LDHA, and HIF-1α (Fig. 5D–H). These results suggested that glycolysis was imperative for PEDV replication.

Considering that PEDV infection enhanced glucose uptake in ST cells (Fig. 1D and E), we next assessed whether glucose availability modulated viral replication. Exposure of PEDV-infected cells to high-glucose conditions (25 mM) resulted in significantly higher mRNA levels of PEDV N compared with low-glucose conditions (5 mM) (Fig. S4E). Consistently, protein expression of PEDV N, HK2, LDHA, and HIF-1α was markedly increased under high-glucose conditions (Fig. 5I–M).

Taken together, these results indicated that glycolysis was essential for PEDV replication and that elevated glucose availability further enhanced viral replication, in line with the increased glycolytic activity observed during PEDV infection.

### 3.6 Glycolysis induced by PEDV infection suppresses the generation of IFNs

**Fig. 6:**
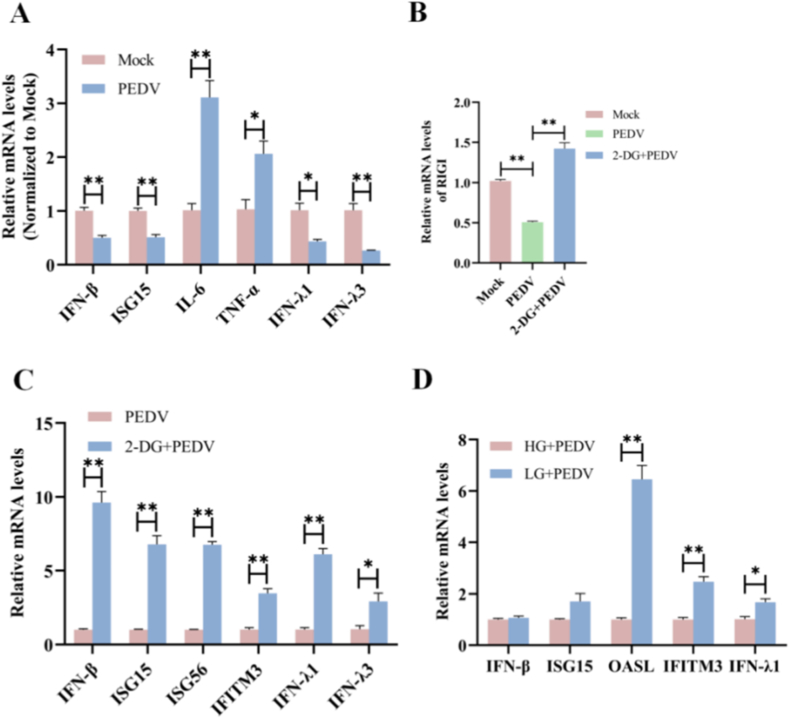
Glycolysis induced by PEDV infection suppresses the generation of IFNs. (A) ST cells were infected with PEDV for 24 h. The mRNA levels of IFNs, ISGs, and inflammatory cytokines were detected by qRT-PCR. (B–C) ST cells were treated with 2-DG for 4 h prior to PEDV infection for 24 h. The mRNA levels of RIG-I (B), IFNs, and ISGs (C) were detected by qRT-PCR. (D) ST cells were exposed to different glucose concentrations (high: 25 mM or low: 5 mM) for 4 h and then infected with PEDV for 24 h. The mRNA levels of IFNs and ISGs were detected by qRT-PCR. Data were presented as mean ± SEM from three independent experiments. ∗*P* < 0.05, ∗∗*P* < 0.01.

Viral infection typically triggers cellular innate immune responses, which are prominently characterized by the induction of IFNs and interferon-stimulated genes (ISGs) to restrict viral replication. To assess the impact of PEDV infection on innate immune signaling, we analyzed the expression of IFNs, ISGs, and inflammatory cytokines by qRT-PCR. The results showed a significant downregulation in mRNA expression of type I and type III IFNs, including IFN-β, IFN-λ1, IFN-λ3, and ISG15, whereas the mRNA expression of proinflammatory cytokines, like IL-6 and TNF-α, exhibited significant upregulation (Fig. 6A). The findings demonstrated that PEDV infection suppressed antiviral interferon responses while enhancing inflammatory signaling.

Considering the reshaped metabolism toward aerobic glycolysis upon PEDV infection, we further examined whether glycolytic activity contributed to the observed suppression of IFNs. Pharmacological inhibition of glycolysis with 2-DG significantly restored the expression of key innate immune signaling molecules, including RIG-I, IFN-β, IFN-λ1, IFN-λ3, and multiple ISGs (ISG15, ISG56, and IFITM3), in PEDV-infected cells (Fig. 6B and C). To further evaluate the role of glucose availability in regulating antiviral immunity, PEDV-infected ST cells were cultured under low- or high-glucose conditions. Cells maintained under low-glucose conditions exhibited significantly higher expression levels of IFN-β, IFN-λ1, ISG15, OASL, and IFITM3 compared with those cultured under high-glucose conditions (Fig. 6D).

Collectively, these findings suggested that aerobic glycolysis induced by PEDV infection impeded type I and type III IFNs responses, while favoring a proinflammatory transcriptional profile, thereby contributing to viral immune evasion.

### 3.7 PEDV-induced lactate production inhibits IFNs responses and facilitates viral replication

**Fig. 7:**
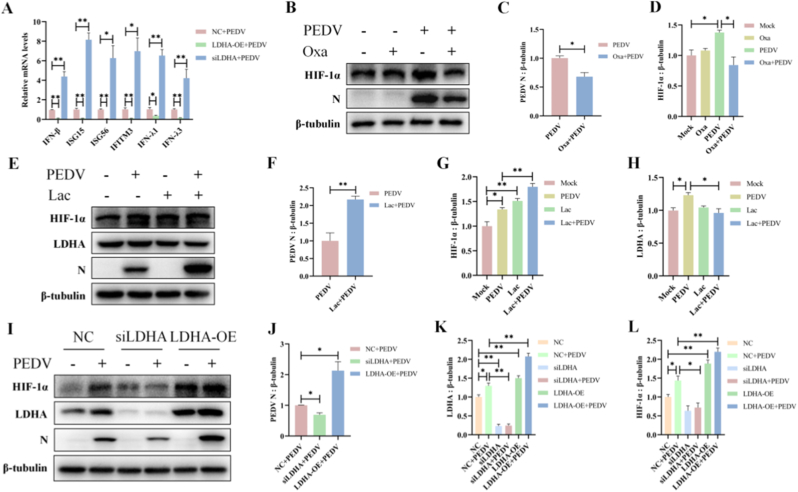
PEDV-induced lactate production inhibits IFNs responses and facilitates viral replication. (A) ST cells were transfected with siLDHA or LDHA-OE for 24 h, and then infected with PEDV for 24 h. The mRNA levels of type I IFNs (IFNβ), type III IFNs ( IFN-λ1, IFN-λ3), and ISGs (IFITM3, ISG-15, ISG-56) were detected by qRT-PCR. (B–D) ST cells were treated with the LDHA inhibitor Oxa, and then infected with PEDV for 24 h. The expression of PEDV N and HIF-1α was detected by western blots. (E–H) ST cells were treated with exogenous lactate (Lac), and then infected with PEDV for 24 h. The expression of PEDV N, HIF-1α and LDHA was detected by western blots. (I–L) ST cells were transfected with siLDHA or LDHA-OE for 24 h, and then infected with PEDV for 24 h. The expression of PEDV N, HIF-1α, and LDHA was detected by western blots. Data were presented as mean ± SEM from three independent experiments. ∗*P* < 0.05, ∗∗*P* < 0.01.

Lactate production catalyzed by LDHA represents a key metabolic outcome of enhanced glycolysis and has been implicated in the regulation of innate immune signaling. To determine whether lactate contributed to the suppression of IFNs during PEDV infection, we modulated LDHA expression and examined the resulting effects on IFNs responses. Overexpression of LDHA significantly inhibited the mRNA expression of type I IFN (IFNβ), type III IFNs (IFN-λ1, IFN-λ3), and ISGs (IFITM3, ISG-15, ISG-56) in PEDV-infected cells, whereas silencing of LDHA treatment markedly restored the expression of these genes (Fig. 7A). These results indicated that lactate production following PEDV infection was negatively correlated with the IFNs responses.

We next examined the effects of altered lactate levels on viral replication. Inhibition of LDHA using the specific inhibitor oxamate significantly reduced the mRNA and protein expression of PEDV N, accompanied by decreased HIF-1α levels (Fig. 7B–D and Fig. S5A and C). In contrast, exogenous lactate treatment markedly enhanced PEDV N and HIF-1α expression at both the transcript and protein levels (Fig. 7E–H and Fig. S5B and C). Similarly, silencing of LDHA by siLDHA significantly suppressed PEDV N and HIF-1α expression, whereas overexpression of LDHA led to a pronounced upregulation in their expression (Fig. 7I–L and Fig. S5D and E).

**Fig. 8:**
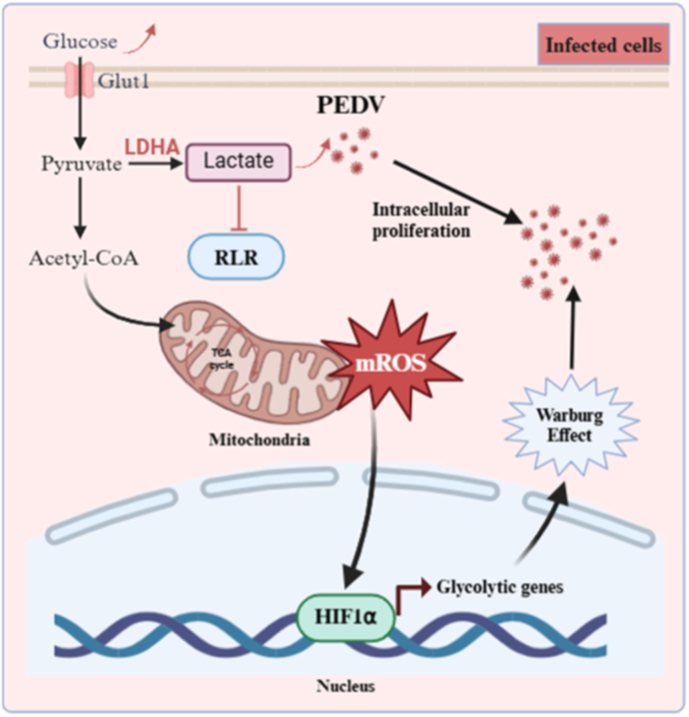
Schematic overview of PEDV-induced glycolytic reprogramming that supports viral replication. PEDV infection induces mitochondrial dysfunction and mROS accumulation, leading to stabilization of HIF-1α, enhanced glycolytic flux, and increased lactate production, which suppresses interferon responses and favors viral replication.

Collectively, these data demonstrate that PEDV-induced lactate accumulation functions as an immunometabolic effector that suppresses the antiviral innate immunity responses, thereby creating a metabolic environment that favors viral replication (Fig. 8).

## 4 Discussion

Recurrent outbreaks of PED continue to pose major challenges to the swine industry, highlighting the need to identify host mechanisms that support PEDV replication and immune evasion for novel antiviral strategies. In the present study, we demonstrated that PEDV infection induced a coordinated metabolic reprogramming in host cells, characterized with enhanced glucose uptake, pronounced accumulation of lactate, and suppression of mitochondrial TCA cycle activity. These are hallmark features of the “Warburg effect”. Although oxidative phosphorylation (OXPHOS) yields more ATP per glucose molecule, glycolysis provides a faster means of energy production and supplies biosynthetic intermediates required for macromolecule synthesis [32]. The observed metabolism shift in PEDV-infected cells toward glycolysis rather than OXPHOS is therefore consistent with the biomolecular fuel and energy demands required for PEDV to sustain its rapid proliferation. Such glycolytic reprogramming has been observed in multiple viral infections, including SARS-CoV-2 [23,24], Marek's disease virus [33], hepatitis C virus [11], and influenza A virus [34], where accelerated glycolysis provides rapid ATP generation and biosynthetic intermediates to support viral replication. We further ovserved that PEDV replication was highly sensitive to glucose availability and glycolytic activity, as pharmacological inhibition of glycolysis or genetic interference with key glycolytic enzymes (HK2) significantly impaired viral replication. This was consistent with previous reports that glucose and glutamine metabolism significantly influenced PEDV replication, and that depletion of key energy substrates inhibited viral proliferation [35,36]. However, it has been reported that Vaccinia virus does not activate glycolysis, and it requires glutamine (Gln) rather than glucose for efficient replication [17], suggesting that viruses may employ unique strategies in regulating cellular metabolism. Collectively, the finding that restricting glycolysis or glucose availability could effectively inhibit PEDV proliferation provided new insights into the development of broad-spectrum antiviral therapeutics against PEDV.

HIF-1α, the oxygen-sensitive subunit of the HIF-1 transcriptional complex, is a well-established regulator of cellular metabolism that integrates environmental and intracellular stress signals to control glycolytic gene expression [37]. In the present study, we observed that PEDV infection markedly activated HIF-1α, and that interference with HIF-1α signaling significantly impaired viral replication, supporting a functional requirement for HIF-1α during PEDV infection. The expression of HIF-1α target genes involved in glucose uptake and glycolysis, including GLUT1, HK2, and LDHA, was upregulated in PEDV-infected cells, consistent with the reinforcement of a glycolytic metabolic program. Intriguingly, the exploitation of HIF-1α-dependent metabolic pathways has also been documented in multiple viral infections [9,[22], [23], [24], [25],^38^]. For example, porcine reproductive and respiratory syndrome virus (PRRSV) nsp1β protein enhances viral replication by stabilizing HIF-1α [25], and the avian reovirus σA protein promotes viral replication through activation of the mTORC1/eIF4E/HIF-1α signaling axis, which coordinates metabolic reprogramming in infected cells [38]. In this context, although the specific PEDV-encoded proteins responsible for HIF-1α activation remain to be identified, our data highlight HIF-1α as a critical host factor exploited by PEDV to sustain metabolic remodeling and viral persistence.

The metabolic shift induced by PEDV infection was accompanied by a pronounced decline in cellular energy homeostasis and mitochondrial integrity. We observed reduced ATP production, altered mitochondrial ultrastructure, and a switch from mitochondrial fusion toward fission, collectively indicating profound mitochondrial dysfunction. Consistently, a recent study also demonstrated that PEDV suppressed mitochondrial respiratory chain activity, promoted mitochondrial depolarization, and drove excessive mtROS accumulation, thereby facilitating viral infection and intestinal pathology [39]. mROS accumulation during PEDV infection was required for HIF-1α stabilization and glycolytic gene upregulation, and that pharmacological scavenging of ROS significantly attenuated glycolytic activation and viral replication, underscoring the functional importance of the mROS–HIF–1α axis during PEDV infection. Previous work has also reported that SARS-CoV-2 ORF3 induced the production of mROS, which stabilized the expression of HIF-1α and subsequently promoted cellular glycolysis and viral replication in monocytes and endothelial cells [24]. Similarly, HCV stabilized HIF-1α via ROS to enhance lipogenesis [11]. Taken together, these findings suggested that mitochondrial dysfunction was not merely a passive consequence of PEDV infection but functioned as a regulatory event that reshaped host metabolic and signaling networks. The accumulation of mROS contributed to HIF-1α stabilization and metabolic reprogramming during PEDV infection. Intriguingly, the severity of SARS-CoV-2 infection in mice can be dramatically reduced by treatment with the mitochondrially targeted catalytic antioxidant drug [40]. Collectively, these findings suggested that reducing mROS may serve as an effective strategy for combating PEDV infection.

Metabolic remodeling during PEDV infection also profoundly impacted innate immune responses. We observed significant suppression of type I and type III IFNs expression alongside enhanced inflammatory cytokine production. These were consistent with previous reports that PEDV antagonized the innate immunity and induced an acute inflammatory response [30,41]. Inhibition of glycolysis or restriction of glucose accessibility (e.g., a low-glucose condition) could restored interferon responses, indicating a functional link between metabolic state and antiviral immunity. Moreover, PEDV-induced metabolic remodeling resulted in significant downregulation of multiple ISGs, several of which have been reported to depend on OXPHOS for sustained expression [42]. These observations suggest that enhanced glycolysis coupled with suppression of OXPHOS may synergistically impair antiviral effector functions, thereby creating an immunosuppressive niche favorable for viral persistence. In addition, lactate, a major by-product of glycolysis, has also been shown to influence viral replication by regulating cytokines secretion [26]. Our results showed that lactate accumulation during PEDV infection inhibited the production of antiviral molecules such as IFNs and ISGs and thus promoted viral replication. Consistently, another study aimed at improving PEDV amplification efficiency and providing high-quality antigens for vaccine preparation also demonstrated that lactate, as a downstream metabolite, was beneficial for the efficient amplification of PEDV in cell culture [35]. However, they did not further elucidate whether the promotion of lactate on PEDV proliferation was associated with the resulting immunosuppressive microenvironment. Particularly noteworthy, lactate has recently emerged as an endogenous regulator of innate immunity, capable of suppressing RIG-I-MAVS signaling and IFNs production in multiple viral infections [26,28]. Similar viral escape strategies have been reported in severe COVID-19 patients upon SARS-CoV-2 infection, correlating with elevated lactate levels and delayed IFN responses [43]. However, whether this represents a conserved strategy for PEDV or other coronaviruses to antagonize innate immunity warrants further investigation.

Notably, in contrast to the metabolic reprogramming strategies and the consequent immunosuppressive niche induced by PEDV or SARS-CoV-2, recent work on porcine deltacoronavirus (PdCoV) revealed a non-Warburg metabolic remodeling characterized by reduced glycolytic flux and lactate production, with pyruvate serving as a central metabolic hub to support nonessential amino acid and nucleotide biosynthesis [44]. This divergence highlights the metabolic heterogeneity among coronaviruses and may reflect the differences in virus replication kinetics, host cell tropisms, or the engagement of upstream viral proteins that initiate metabolic remodeling. Taken together, these findings suggest that PEDV preferentially amplifies a glycolytic-lactate axis that directly couples metabolic reprogramming to innate immune suppression, thereby integrating metabolic supply with immune evasion.

In this study, however, several limitations should be acknowledged. The experiments herein were mainly performed in ST cells, and validation in primary porcine intestinal epithelial cells, intestinal organoids or *in vivo* infection models will be essential to confirm physiological relevance in future. In addition, although our analyses captured key metabolic alterations at representative infection conditions, a comprehensive characterization of metabolic dynamics across different MOIs and time points was not performed. As part of our future work, we will systematically compare the temporal and dose-dependent metabolic regulation during PEDV infection in primary porcine intestinal epithelial cells, intestinal organoids, and, where feasible, *in vivo* infection models, to better mirror natural infection levels and further validate the impact of metabolic interventions on PEDV replication. Moreover, while pharmacological inhibition enabled us to define key host redox-metabolic nodes that regulated PEDV replication, the upstream viral determinants responsible for initiating mitochondrial dysfunction and activating the ROS/HIF-1α axis were not identified in the present study. Future work employing targeted overexpression or knockdown of individual PEDV-encoded proteins will be important to delineate the potential viral factors linking PEDV infection to mitochondrial impairment and metabolic remodeling, thereby further elucidating the molecular mechanisms underlying PEDV pathogenesis.

In conclusion, this study demonstrated that PEDV infection drove a coordinated metabolic reprogramming in host cells, characterized by a shift toward glycolytic metabolism. Mechanistically, PEDV-induced mitochondrial dysfunction and excessive mROS accumulation stabilized HIF-1α, leading to sustained upregulation of glycolytic genes and establishment of a metabolic circuit that supported viral replication. This metabolic shift not only supported the bioenergetic and biosynthetic demands of viral replication but also contributed to an immunosuppressive intracellular environment through lactate accumulation, thereby attenuating antiviral responses and facilitating efficient PEDV propagation. Together, our work identifies a metabolic-immune regulatory framework exploited by PEDV and provides a mechanistic basis for the rational development of metabolism-focused antiviral strategies.

## CRediT authorship contribution statement

**Yafang Xu:** Methodology, Project administration, Validation, Visualization. **Jinqiu Zhang:** Conceptualization, Funding acquisition, Project administration, Supervision, Writing – original draft, Writing – review & editing. **Chengwei Yin:** Formal analysis, Software. **Laizhen Liu:** Investigation, Methodology. **Zhenglei Wang:** Resources. **Shaodong Fu:** Formal analysis, Methodology. **Rong Fan:** Validation, Visualization. **Yanyan Zhao:** Software, Visualization. **Jinfeng Miao:** Supervision, Writing – original draft, Writing – review & editing.

## Declaration of competing interest

The authors declare that they have no conflicts of interest with the contents of this article.

## Data Availability

Data will be made available on request.
