# Supplementary material for: Porcine epidemic diarrhea virus promotes viral replication via ROS/HIF-1α-mediated glycolysis
Source: Redox Biol. 2026 Jan 5;89:104008. doi: 10.1016/j.redox.2026.104008 (PMC12814091; doi:10.1016/j.redox.2026.104008)
Supplement: Multimedia component 1 [file mmc1.docx]

**Table S1: Sequences of qRT-PCR primers and siRNAs**

| Names | Primer | Sequence (5’-3’) |
| --- | --- | --- |
| GLUT1 | Forward | CACTGTCGTGTCGCTGTTC |
|  | Reverse | ATGCTCAGGTAGGACATCCAG |
| HK2 | Forward | CCTTGGCCCGCTGAGATAA |
|  | Reverse | GAATCTGGCCACGGGGATTA |
| PFK | Forward | ACGAGAAGTGCCACGAATAC |
|  | Reverse | GGAGGAGTACAGGTTGTAGAGA |
| PGK1 | Forward | GCTGGACGTGAAGGGAAAGA |
|  | Reverse | CTGACTTGGCTCCGTTGTCT |
| PKM | Forward | CTCAGCATCTTCTCACCCATC |
|  | Reverse | GTAGCCAAACACCTCCACTT |
| LDHA | Forward | TGCCTGTATGGAGCGGAGTA |
|  | Reverse | TGCCACAGATAGTCCAATGGC |
| IDH3G | Forward | AAAACATGCACACGCCAGAC |
|  | Reverse | AGCGGGTTTATTCTGGGTCC |
| OGDH | Forward | AGTGAGAACGGAGTGGACTA |
|  | Reverse | TTGAGTCGAACTGGCAGAAG |
| SDHB | Forward | CAAAAATCTACCCTCTTCCACA |
|  | Reverse | CTTGTCTCCATTCCACCAGTAG |
| PEDV N | Forward | GGAGTCGTGGTAATGGCAAC |
|  | Reverse | CTCTGTTCTGGGAAGCTCCA |
| β-actin | Forward | CTTAGTTGCGTTACACCCTTTC |
|  | Reverse | TGTCACCTTCACCGTTCCA |
| HIF-1α | Forward | GGCGCGAACGACAAGAAAAA |
|  | Reverse | GTGGCAACTGATGAGCAAGC |
| IFN-β | Forward | CCACCACAGCTCTTTCCATGA |
|  | Reverse | TGAGGAGTCCCAGGCAACT |
| ISG15 | Forward | GGTGAGGAACGACAAGGGTC |
|  | Reverse | GGCTTGAGGTCATACTCCCC |
| OASL | Forward | TCCCTGGGAAGAATGTGCAG |
|  | Reverse | CCCTGGCAAGAGCATAGTGT |
| IFITM3 | Forward | GCTGCCCTTCCACCAACG |
|  | Reverse | ACTGAGTCGATCATCCTTCTCC |
| ISG56 | Forward | AAATGAATGAAGCCCTGGAGTATT |
|  | Reverse | AGGGATCAAGTCCCACAGATTTT |
| IL-6 | Forward | GCTGCAGTCACAGAACGAGT |
|  | Reverse | CAGGTGCCCCAGCTACATTA |
| TNF-α | Forward | ACGCTCTTCTGCCTACTGC |
|  | Reverse | TCCCTCGGCTTTGACATT |
| IFN-λ1 | Forward | CCACGTCGAACTTCAGGCTT |
|  | Reverse | ATGTGCAAGTCTCCACTGGT |
| IFN-λ3 | Forward | GCCAAGGATGCCTTTGAAGAG |
|  | Reverse | CAGGACGCTGAGGGTCAGG |
| RIG-I | Forward | TTTCCACGAGGACGAAAGGG |
|  | Reverse | GTGTCAGACGTTGCTCCAGA |
| siNC | Forward | UUCUCCGAACGUGUCACGUTT |
|  | Reverse | ACGUGACACGUUCGGAGAATT |
| siHK2 | Forward | GCCACUUUGAGACCAAAGATT |
|  | Reverse | UCUUUGGUCUCAAAGUGGCTT |
| siLDHA | Forward | CCGAUUUCCACCAUGAUUATT |
|  | Reverse | UAAUCAUGGUGGAAAUCGGTT |

**Supplementary Figure 1**

Fig. S1. PEDV infection reprograms host metabolism toward aerobic glycolysis. ST cells were infected with PEDV JS2013 (MOI=1) for 24 h. The expression of metabolites associated with glycolysis (A) and citrate cycle (B) and key enzymes in the glycolytic pathway (C) was determined by qRT-PCR. Data were shown as mean ± SEM of three independent experiments. * *P* < 0.05, ** *P* < 0.01.

**Supplementary Figure 2**

Fig. S2. Upregulation of HIF-1α after PEDV infection. ST cells were infected with PEDV JS2013 (MOI=1) for 24 h. The mRNA expression of HIF-1α was determined by qRT-PCR. Data were shown as mean ± SEM of three independent experiments. * *P* < 0.05, ** *P* < 0.01.

**Supplementary Figures 3**

Fig. S3. Mitochondrial mROS-mediated HIF-1α stabilization upon PEDV infection favors viral replication. (A-B) ST cells were treated with or without NAC for 4 h, then cells were infected with or without PEDV (MOI= 1) and mROS were detected using flow cytometer. (C-E) The mRNA levels of N, HIF-1α, and HK2 gene levels were detected by qRT-PCR. Data were shown as mean ± SEM of three independent experiments. * *P* < 0.05, ** *P* < 0.01.

**Supplementary Figures 4**


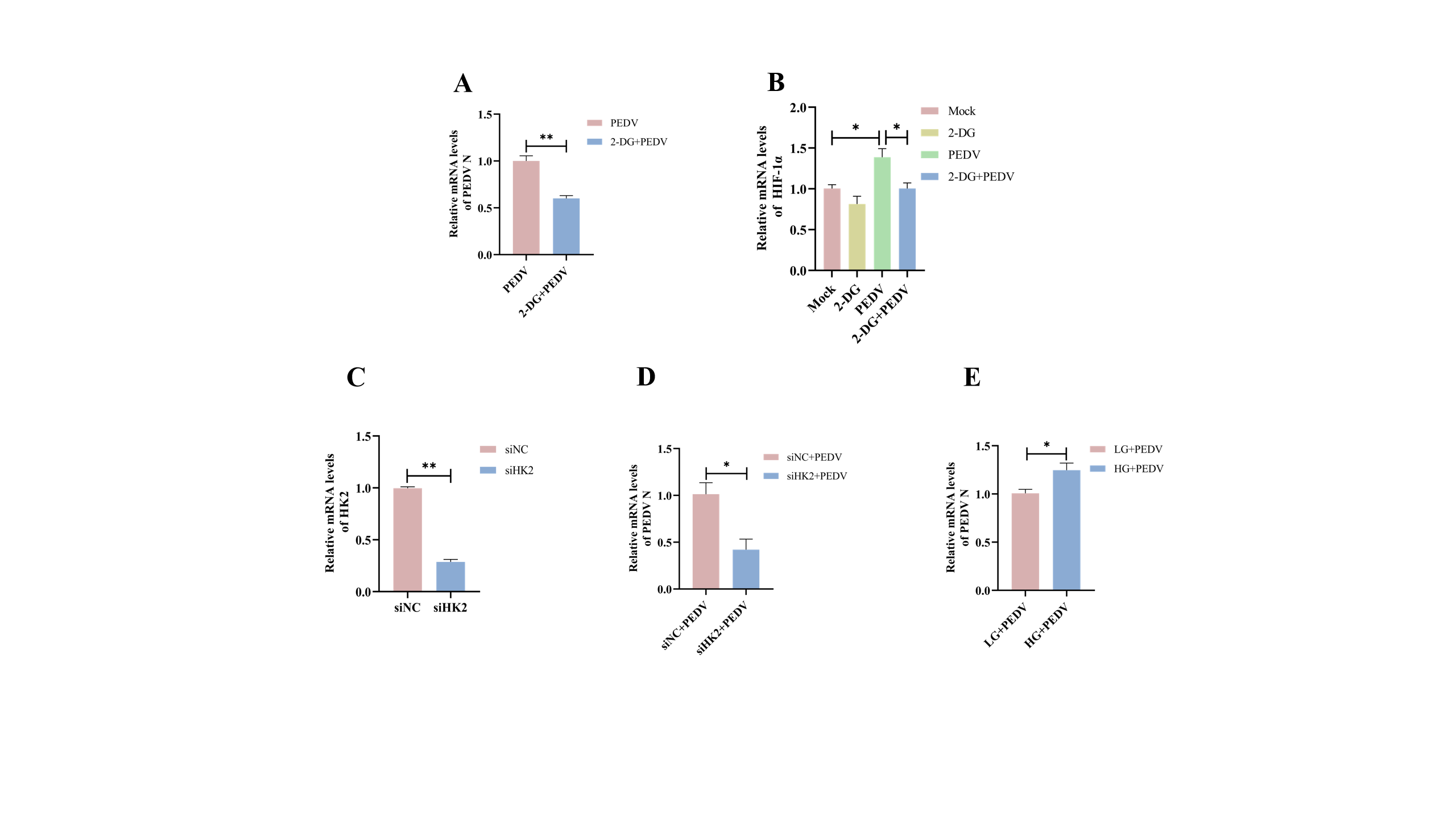


Fig. S4. Glycolysis is essential for PEDV replication and elevated glucose levels enhance PEDV replication. (A and B) ST cells were treated with or without 2-DG for 4 h, then infected with PEDV at an MOI of 1 and incubated in the presence or absence of 2-DG for 24 h. The mRNA levels of N, HIF-1α gene were analyzed by qRT-PCR. (C) HK2 knockdown by siRNA was confirmed by qRT-PCR. (D) ST cells were transfected with siHK2 for 24 h, and then infected with PEDV at an MOI of 1 for 24 h. The mRNA level of N gene was analyzed by qRT-PCR. (E) ST cells were exposed to different concentrations of glucose (high: 25 mM or low: 5 mM) for 4 h, then infected with PEDV at an MOI of 1, and incubated in different concentrations of glucose (high: 25 mM or low: 5 mM) for 24 h. The mRNA level of N gene was analyzed by qRT-PCR. Data were shown as mean ± SEM of three independent experiments. * *P* < 0.05, ** *P* < 0.01.

**Supplementary Figures 5**


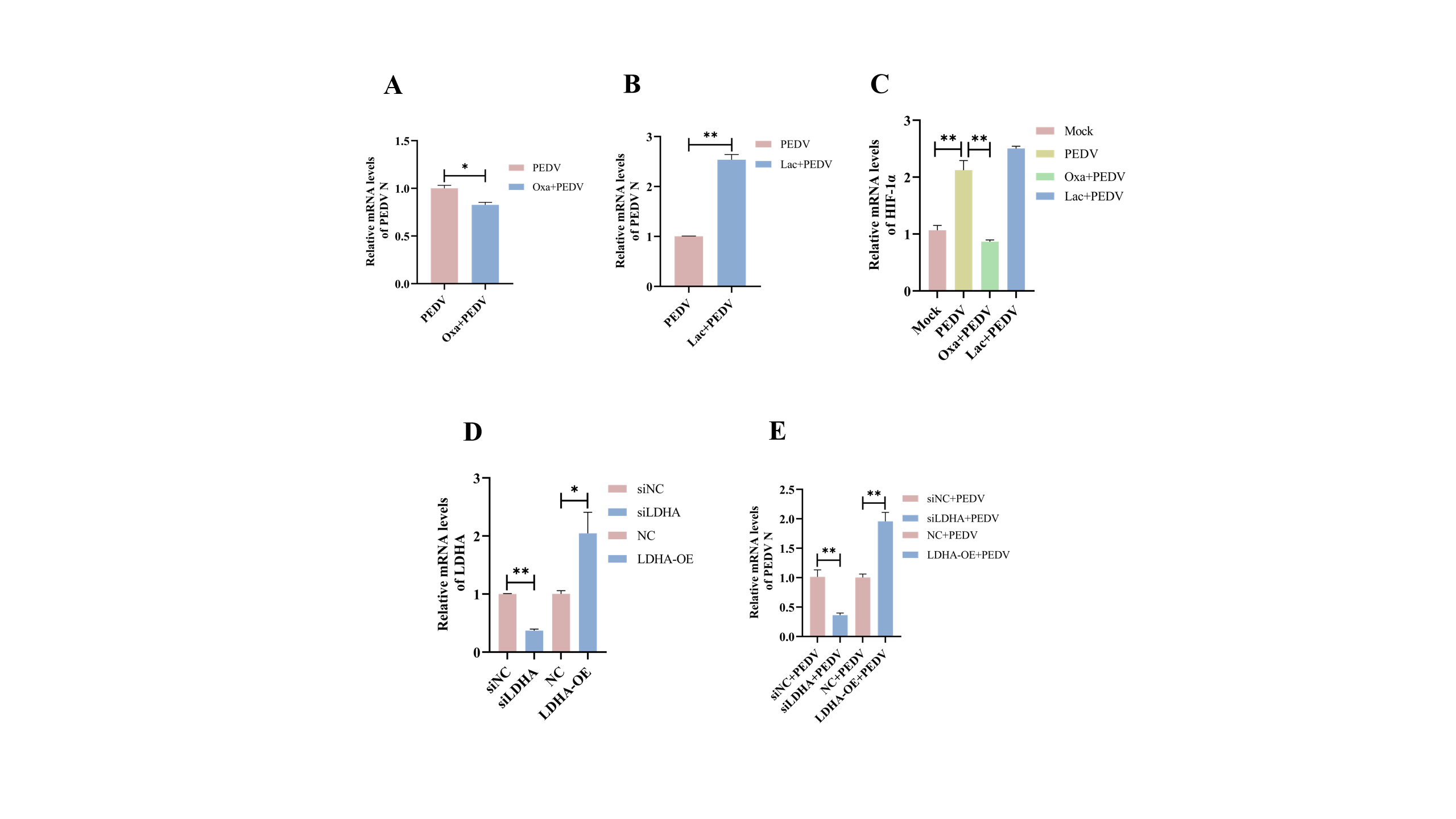


Fig. S5. PEDV infection promotes lactate production to inhibit IFNs and facilitate viral replication. (A-C) ST cells were infected with PEDV at an MOI of 1 and incubated in the presence or absence of Lac or Oxa. Cells were harvested at 24 hpi. The mRNA levels of N and HIF-1α were analyzed by qRT-PCR. (D) ST cells were transfected with siLDHA or treated with LDHA-OE for 24 h, and intracellular mRNA levels of LDHA gene were analyzed by qRT-PCR. (E) ST cells were transfected with siLDHA or treated with LDHA-OE for 24 h, then infected with PEDV at an MOI of 1 for 24 h. The mRNA level of N gene was analyzed by qRT-PCR. Data were shown as mean ± SEM of three independent experiments. * *P* < 0.05, ** *P* < 0.01.

**Supplementary Figures 6**

Fig. S6. The replication of PEDV JS2013 in different cell lines. (A) The cytopathic effect of Vero, IPEC-J2 and ST cells after PEDV infection. Scale bar = 275 μm. (B) The growth curves of PEDV JS2013 in different cell lines were determined by measuring the virus titers at different time points after viral infection. (C) ST cells were infected with PEDV JS2013 at an MOI of 1. The expression of PEDV N protein was determined by Western blot. (D) ST cells were infected with PEDV JS2013 at different MOIs, and PEDV N protein at 24 hpi was analyzed by Western blot. (E) The replication of PEDV JS2013 in ST cells at 24 hpi was detected by IFA. Scale bar = 10 μm. Data were shown as mean ± SEM of three independent experiments. * *P* < 0.05, ** *P* < 0.01.

**Supplementary Figure 7**


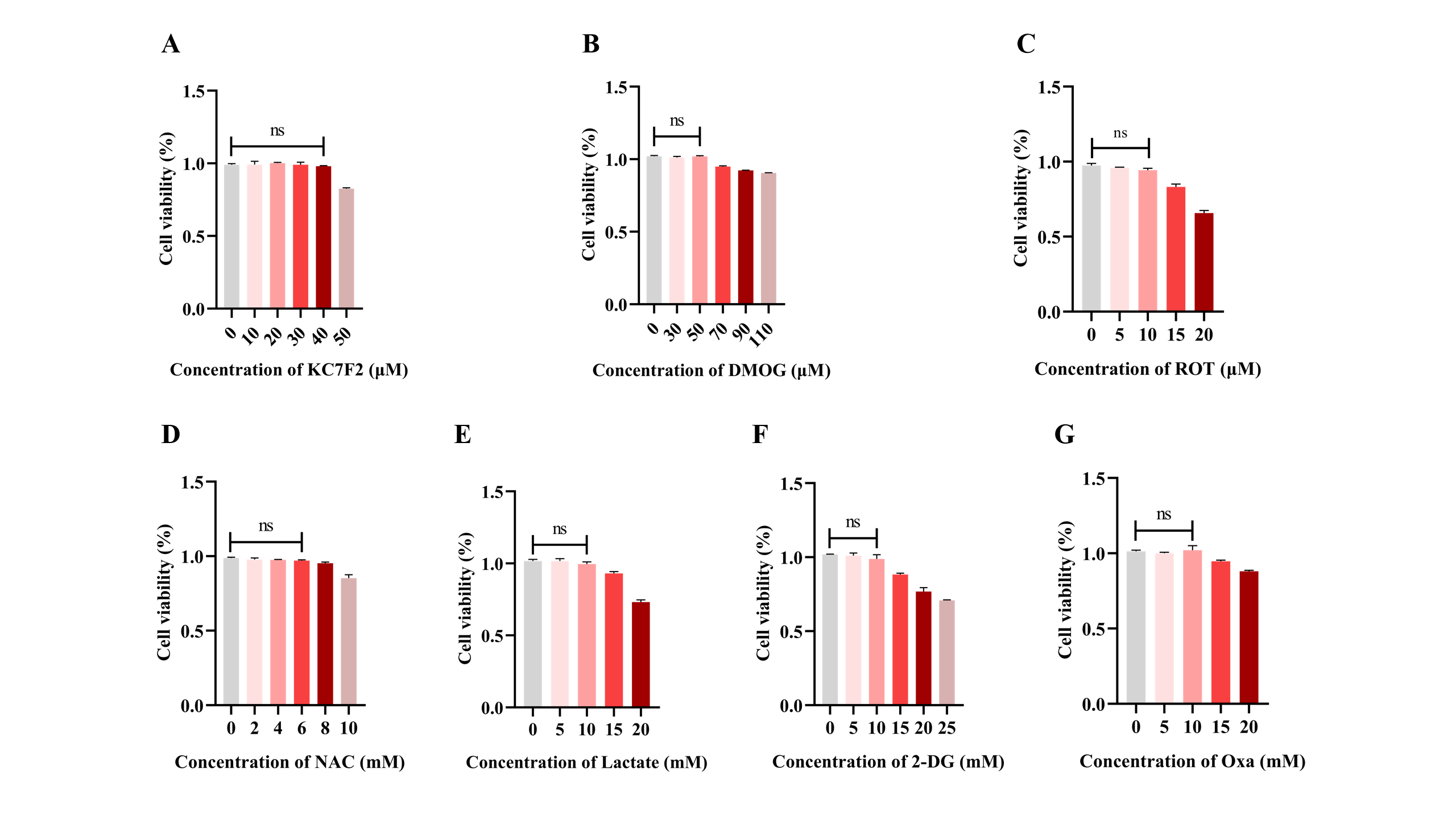


Fig. S7. Cell viability assay. ST cells were treated with KC7F2 for 24 h (A), DMOG for 24 h (B), ROT for 1 h (C), NAC for 4 h (D), lactate for 4 h (E), 2-DG for 4 h (F), and Oxa for 4 h (G) at different concentrations, respectively, and the cell viability was analysized by CCK-8 assay.

**Supplementary Figure 8**


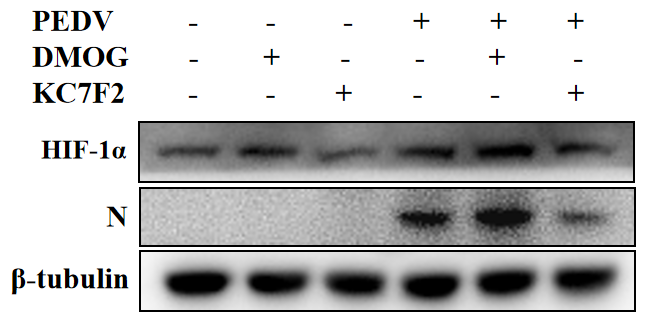


Fig. S8. ST cells were treated with 40 μM HIF-1α inhibitor KC7F2 or 50 μM HIF-1α activator DMOG, respectively, for 24 h, and then infected with or without PEDV for 24 h. The expressions of HIF-1α, PEDV N were determined by western blots.
